# HIV-1 Envelope Resistance to Proteasomal Cleavage: Implications for Vaccine Induced Immune Responses

**DOI:** 10.1371/journal.pone.0042579

**Published:** 2012-08-06

**Authors:** Nicholas J. Steers, Silvia Ratto-Kim, Mark S. de Souza, Jeffrey R. Currier, Jerome H. Kim, Nelson L. Michael, Carl R. Alving, Mangala Rao

**Affiliations:** 1 United States Military HIV Research Program, Walter Reed Army Institute of Research, Silver Spring, Maryland, United States of America; 2 Henry M Jackson Foundation for the Advancement of Military Medicine, Bethesda, Maryland, United States of America; 3 Armed Forces Research Institute for Medical Sciences, Bangkok, Thailand; Massachusetts General Hospital, United States of America

## Abstract

**Background:**

Antigen processing involves many proteolytic enzymes such as proteasomes and cathepsins. The processed antigen is then presented on the cell surface bound to either MHC class I or class II molecules and induces/interacts with antigen-specific CD8+ and CD4+ T-cells, respectively. Preliminary immunological data from the RV144 phase III trial indicated that the immune responses were biased towards the Env antigen with a dominant CD4+ T-cell response.

**Methods:**

In this study, we examined the susceptibility of HIV-1 Env-A244 gp120 protein, one of the protein boost subunits of the RV144 Phase III vaccine trial, to proteasomes and cathepsins and identified the generated peptide epitope repertoire by mass spectrometry. The peptide fragments were tested for cytokine production in CD4^+^ T-cell lines derived from RV144 volunteers.

**Results:**

Env-A244 was resistant to proteasomes, thus diminishing the possibility of the generation of class I epitopes by the classical MHC class I pathway. However, Env-A244 was efficiently cleaved by cathepsins generating peptide arrays identified by mass spectrometry that contained both MHC class I and class II epitopes as reported in the Los Alamos database. Each of the cathepsins generated distinct degradation patterns containing regions of light and dense epitope clusters. The sequence DKKQKVHALF that is part of the V2 loop of gp120 produced by cathepsins induced a polyfunctional cytokine response including the generation of IFN-γ from CD4^+^ T-cell lines-derived from RV144 vaccinees. This sequence is significant since antibodies to the V1/V2-loop region correlated inversely with HIV-1 infection in the RV144 trial.

**Conclusions:**

Based on our results, the susceptibility of Env-A244 to cathepsins and not to proteasomes suggests a possible mechanism for the generation of Env-specific CD4^+^T cell and antibody responses in the RV144 vaccinees.

## Introduction

Peptide-loaded MHC class I molecules are expressed on the surface of all nucleated cells, while MHC class II molecules are expressed on the surface of professional antigen presenting cells. For the clearance of intercellular pathogens during the course of an infection, foreign antigens specific to the pathogen are processed and presented on MHC class I and/or MHC class II molecules [1]–[3]. The presentation of foreign peptides by MHC class I and MHC class II molecules on the surface of cells induce epitope specific CD8^+^ and CD4^+^ T-cells. These antigen-specific T cells are then recalled during re-exposure to the pathogen. Antigen processing and presentation is a complex process involving many proteins working in a defined order. Although there are differences in the proteins required for MHC class I and MHC class II processing and presentation, antigens processed through one pathway can also be presented by the other pathway [4].

MHC class I processing involves many proteins such as ubiquitination proteins, chaperone proteins, loading and transporter proteins, and proteases including the proteasome complex. Endogenous antigens within the cytoplasm are mainly processed by proteasomes [5]–[12] before transportation into the endoplasmic reticulum via the transporter associated with antigen processing. Further trimming of these peptides occurs within the ER before the peptides can be loaded onto the MHC class I molecules [13]. The 8–10 amino acid epitope bound to an MHC class I molecule is then transported to the cell surface.

MHC class II processing of exogenous and endogenous antigens occurs in the endosomal/lysosomal compartment. The antigens can enter the endosomal compartment through endocytosis, phagocytosis, or by autophagy [14]. The antigens are processed by cathepsins and other proteases present in endosomes/lysosomes. There are several cathepsins some of which are cell-type specific. Cathepsins L and S are cysteine proteases while cathepsin D is an aspartic protease. These enzymes cleave endocytosed antigens and generate peptides for MHC class II binding as well as remove the invariant chain chaperone [15], [16]. The processed antigen is presented on the cell surface as a 12–15 amino acid epitope bound to an MHC class II molecule [2], [17]–[20].

Intracellular pathogens have evolved multiple mechanisms to avoid the host's immune response and one principal mechanism is to disrupt or prevent antigen processing and presentation. This can potentially negate or alter the epitope repertoire of foreign epitopes bound to MHC class I or MHC class II molecules on the cell surface [8], [21]–[26]. Apart from directly interacting with the antigen processing and presentation machinery, the biochemical properties of antigens such as disulfide bonds and glycosylation may also influence antigen processing [27], [28]. Disulfide bonds and folding may impact the ability of specific proteases such as the proteasome to proteolytically cleave folded antigens [28]. The processing of exogenous and endogenous glycosylated protein antigens can be impacted by the presence of terminal mannose and fructose residues, which could predetermine the initial processing of glycoprotein antigens within lysosomes and endosomes [29], [30].

The interaction of either mannose or fructose with the appropriate lectins on the cell surface induces the formation of phagosomes and engulfment of the pathogen or antigen for delivery into the phagolysosome for proteolytic degradation and presentation on MHC class I and class II molecules [31]. HIV-1 envelope (Env) is heavily glycosylated with mannose [32], [33], and studies using immature dendritic cells have demonstrated that the envelope protein is capable of being processed in the phagolysosome [34]. Endogenous glycoproteins generated within the cell can be cannibalized by lysosomes through a mechanism known as autophagy [14] and the proteolytically cleaved peptides can be presented by both MHC class I and class II molecules.

Elucidating how antigens are processed and presented is critical for the design of vaccines. The recent RV144 Phase III clinical trial in Thailand using a canary pox vector ALVAC (vCP1521), carrying HIV clade B and circulating recombinant form (CRF)01_AE *gag*, *pro* and *env* genes, and AIDSVAX®B/E (genetically engineered gp120) protein as the vaccine demonstrated a modest efficacy of 31.2% [35]. An analysis for correlates of risk suggested that, among cellular assays, the production of cytokines after stimulation of PBMC from volunteers was inversely correlated with infection rate, although statistically the effect was less robust than the correlates identified for IgG binding to a conformational V1/V2 epitope(s) and Env-specific IgA [36]. Preliminary immunological analysis of RV144 vaccinee samples demonstrated that the vaccine induced a very poor direct ex vivo CD8^+^ T cell response and that both the CD4 cellular and the humoral immune responses were biased towards the HIV-1 gp120 Env antigen. In order to determine if the reason for the low Env-specific CD8^+^ T cell response compared to the CD4^+^ T-cell response was because of the efficiency of antigen processing, this study examined the susceptibility of Env-A244 gp120 protein, one of the components of the bivalent gp120 protein subunit boosts used in the RV144 trial. Env-A244 gp120 protein was subjected to cleavage by purified proteasomes or cathepsins or cathepsins followed by proteasomes and the peptides generated were characterized by mass spectrometry. The peptides generated were examined for their functional activity. We have identified a peptide fragment derived from cathepsins D and K degradations of Env-A244 gp120 (Env-A244) protein containing the sequence DKKQKVHALF in the V2 loop of gp120 that induced a polyfunctional cytokine response including the generation of IFN-γ from CD4^+^ T-cell lines derived from RV144 vaccinees suggesting a possible link between proteolytic processing and induction of vaccine-specific CD4^+^ T helper cells.

## Results

### HIV-1 Env protein is resistant to proteasomal degradation

It is critical to understand how CD4 and CD8-specific T cell epitopes are generated in order to induce the best priming and recall immune responses. Numerous proteases including proteasomes and cathepsins are required to generate antigenic epitopes. In the present study, Env-A244 was chosen for cathepsin and proteasomal degradation, as this antigen was a component of AIDSVAX®B/E used as a boost in the prime-boost RV144 vaccine regimen. HIV-1 Gag-p24 was used as a control protein to demonstrate that the proteasomes used were functionally active [10].

**Figure 1:**
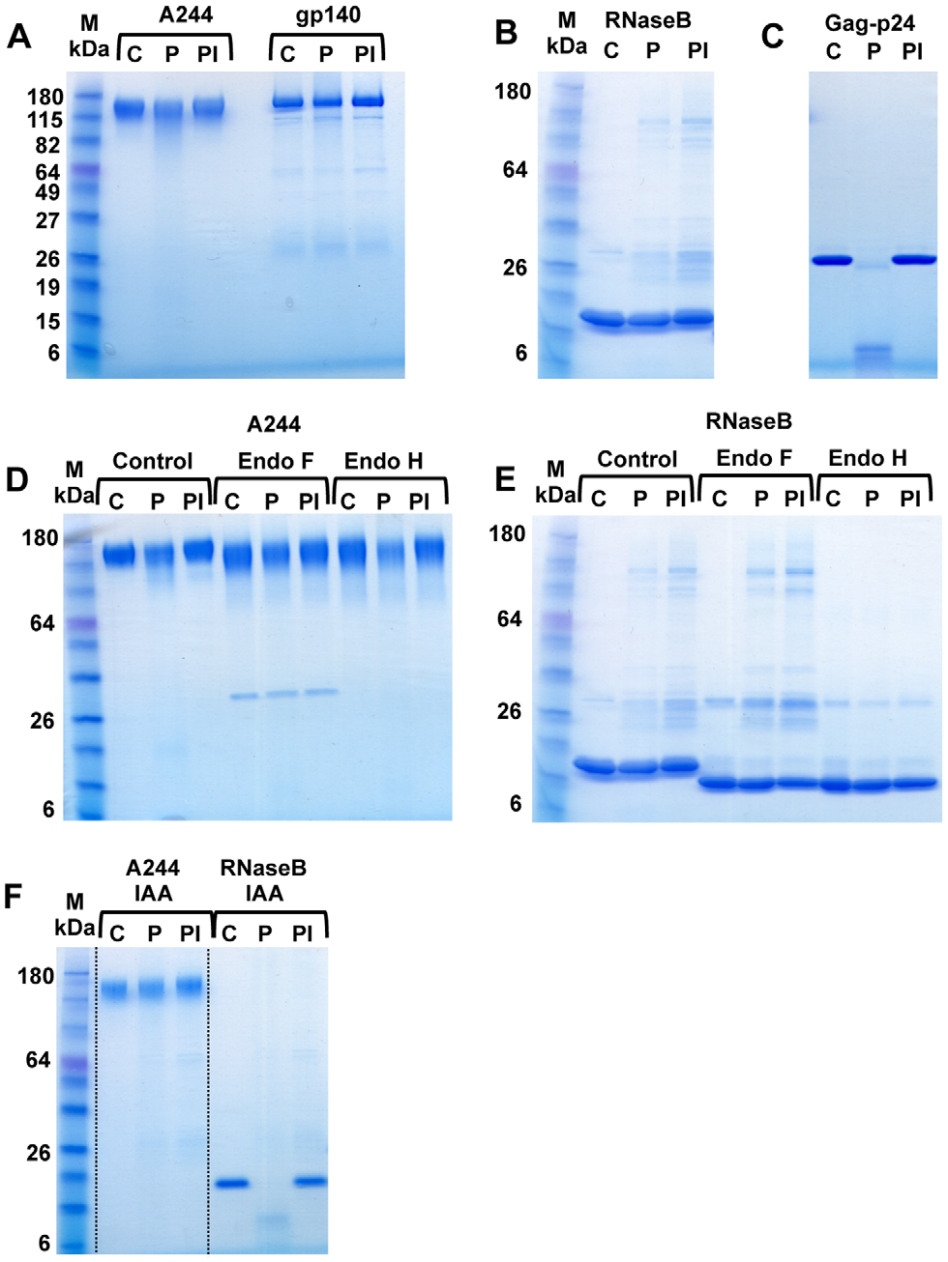
Proteasomes are unable to cleave HIV-1 Env proteins. Env-A244 (CRF01_AE) derived from CHO cells and gp140 (clade B) derived from H9 cells (panel A, lanes C) were treated with purified proteasomes isolated from activated CD4^+^ T-cells in the absence (lanes, P) or presence of epoxomicin, an irreversible proteasome inhibitor (lanes PI). Proteasomes were unable to cleave the Env proteins from two different HIV-1 clades. In panel B, RNaseB, a folded-glycosylated protein (lane C) was treated as in panel A with purified proteasomes in the absence (lane P) or presence of epoxomicin (lane PI). The proteasomes were also unable to cleave RNaseB. The functional activity and the specificity of the proteasomes were demonstrated by the proteolytic cleavage of Gag-p24 (panel C, lane C) in the absence (lane P) or presence of epoxomicin (lane PI). Env-A244 and RNaseB (panels D and E, lanes C) were subjected to the same treatment as in panels A and B. Env-A244 and RNaseB were treated with Endo-F and Endo-H followed by incubation with purified proteasomes in the absence (lanes P) or presence of epoxomicin (lanes PI). Proteasomes were unable to cleave Env-A244 and RNaseB following treatment with either Endo-F or Endo-H. Env-A244 and RNaseB (panel F, lanes C) were reduced and alkylated (IAA) by treatment with iodioacetamine and DTT and then treated with purified proteasomes in the absence (lanes P) or presence of epoxomicin (lanes PI). Env-A244 remained resistant while RNaseB became susceptible to proteasomal cleavage. Samples were run on a 4–20% gradient Tris-glycine polyacrylamide gel. The molecular weight markers are shown in lane M in panels A, B, D, E, and F.

HIV-1 Env-A244 gp120 (CRF01_AE) derived from CHO cells and gp140 (clade B) derived from H9 cells (Figure 1 A, lanes C) were treated with proteasomes (lanes P) purified from activated CD4^+^ T-cells in the absence or presence of proteasome inhibitor (lanes PI), epoxomycin and analyzed on a gradient SDS-polyacrylamide gel. Env-A244 is heavily glycosylated with mannose being one of the principal sugar moieties. Although it has been reported that proteasomes are capable of cleaving glycoproteins [27], [28] proteasomes however, were unable to cleave the glycosylated and folded Env-A244 and gp140 proteins (Figure 1 A). Interestingly, Env-A244 has a gD-tag, and this was effectively cleaved by the proteasomes as identified by mass spectrometry (data not shown). It could be hypothesized that the presence of mannose residues and the folded structure could afford Env-A244 and gp140 a certain degree of protection from proteolytic degradation. This hypothesis is supported by previously published results demonstrating that proteasomes were unable to cleave RNaseB in its native glycosylated and folded form [28]. In our studies also, RNaseB (Figure 1 B, lane C) could not be cleaved by proteasomes (lane P). Again, proteasomes treated with epoxomicin served as a control (lane PI). The lack of RNaseB cleavage by proteasomes was not due to a loss of enzymatic activity as demonstrated by the cleavage of clade B Gag-p24 (Figure 1 C). The specificity of the proteasomal degradation (lane P) of Gag-p24 (lane C) was demonstrated by the lack of proteolytic degradation of Gag-p24 in the presence of the epoxomicin (lane PI).

To determine if the presence of high mannose residues in Env-A244 interfered with proteasomal degradation, Env-A244 (Figure 1 D, lane C) and RNaseB (Figure 1 E, lane C) were treated with Endo-F and Endo-H enzymes and were run under identical conditions as in Figures 1 A and 1 B. Although the high mannose oligosaccharides were removed by Endo-F and Endo-H enzymes as shown by a decrease in the molecular weights of Env-A244 (Figure 1 D, lanes C) and RNaseB (Figure 1 E, lanes C), the proteins still remained resistant to proteasomal cleavage in the absence (Figures 1 D and 1 E, lanes P) or in the presence of epoxomicin (Figures 1 D and 1 E, lanes PI) as determined by SDS-PAGE. Similar results were obtained with gp140 (data not shown). This would suggest that the structure of the protein and not the presence of mannose residues determined the susceptibility to proteasomal degradation. RNaseB contains four disulfide bonds and is resistant to proteasomal degradation until the protein is reduced and unfolded [27], [28]. Reduction and blocking of the cystine residues of RNaseB by treatment with DTT and iodioacetamide (RNaseB-IAA) (Figure 1 F, lane C) enabled the proteasome to effectively degrade RNaseB-IAA (lane P). The specificity of the proteolytic cleavage of RNaseB-IAA was demonstrated by the lack of proteolytic degradation of RNaseB-IAA in the presence of epoxomicin (lane PI). Env-A244 contains multiple disulphide bonds and in contrast to RNaseB, even after treatment with DTT and iodioacetamide (Env-A244-IAA, Figure 1 F, lane C), Env-A244-IAA remained resistant to proteasomal cleavage in the absence (lane P) or presence of epoxomicin (lane PI).

### HIV-1 Env protein is susceptible to cathepsin degradation

**Figure 2:**
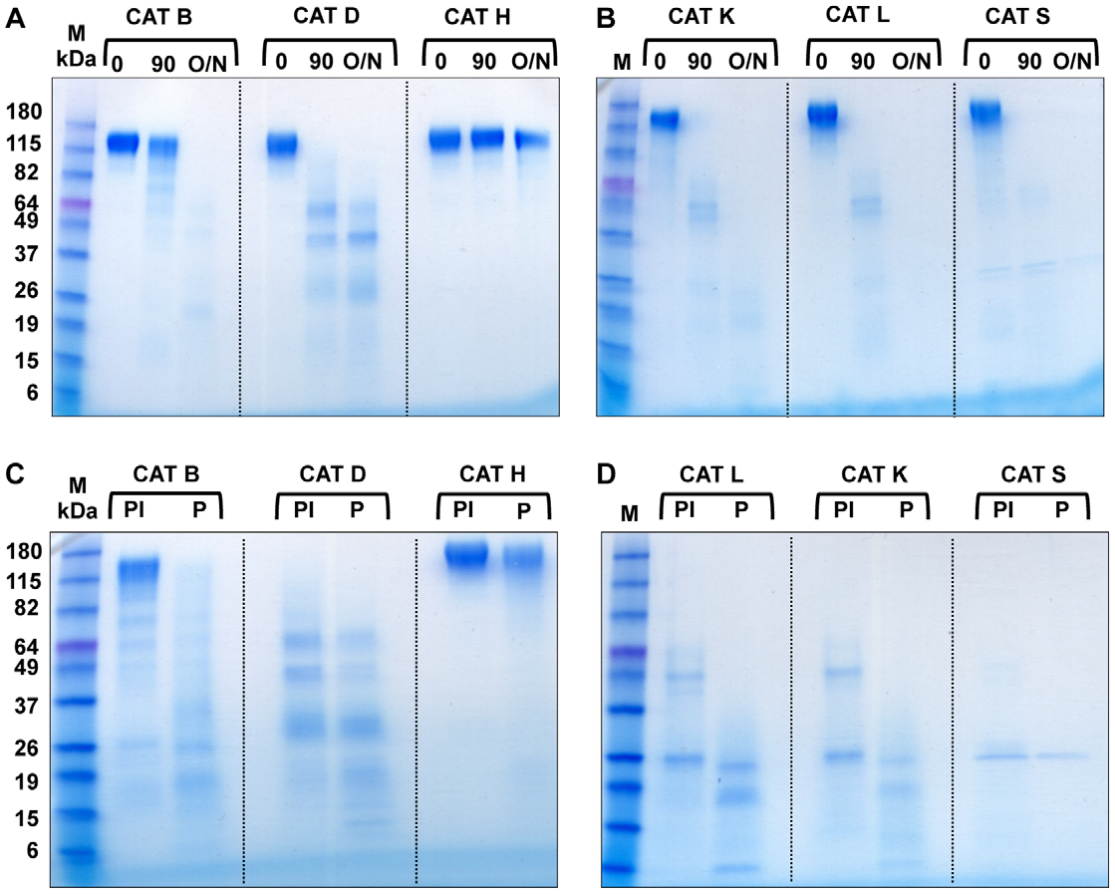
CAT are able to cleave HIV-1 Env-A244 and CAT degradation products are susceptible to proteasomal degradation. HIV-1 Env-A244 (panels A and B, lanes 0) was treated with CAT for 90 min or 16 hrs and then analyzed on a 4–20% gradient Tris-glycine polyacrylamide gel. The 90 min (lanes 90) and 16 hrs (lanes 0/N) degradation pattern with the various CAT are shown in panels A and B. Env-A244 was treated with the individual CAT for 90 min followed by proteasomes in the presence (panels C and D, lanes PI) or absence of epoxomicin (lanes P). The molecular weight markers are shown in lane M in all panels.

Env-A244 was subjected to cathepsin (CAT) degradation (Figures 2 A and 2 B) and then the degradation products were analyzed on a gradient SDS-polyacrylamide gel. The 90 min cathepsin degradation products were then subjected to proteasomal degradation for 16 hrs (Figures 2 C and 2 D). Although Env-A244 (Figures 2 A and 2 B, lanes 0) was resistant to proteasomal degradation (Figure 1 A), the protein was susceptible to CAT B, D, K, L, and S degradation (Figures 2 A and 2 B). The 90 min (lanes 90) and 16 hrs degradation products (lanes O/N) are respectively shown in Figures 2 A and 2 B. The respective CAT degradation products at 90 min were then subjected to proteasomal degradation in the presence (Figures 2 C and 2 D, lanes PI) or absence (lanes P) of epoxomicin. Env A-244 was resistant to cathepsin H degradation at the 90 min as well as at the 16 hrs time point (Figure 2A, lanes 90 and O/N), and as a result remained resistant to proteasomal cleavage in the presence or absence of epoxomicin (Figure 2 C, lanes PI and P, respectively).

**Figure 3:**
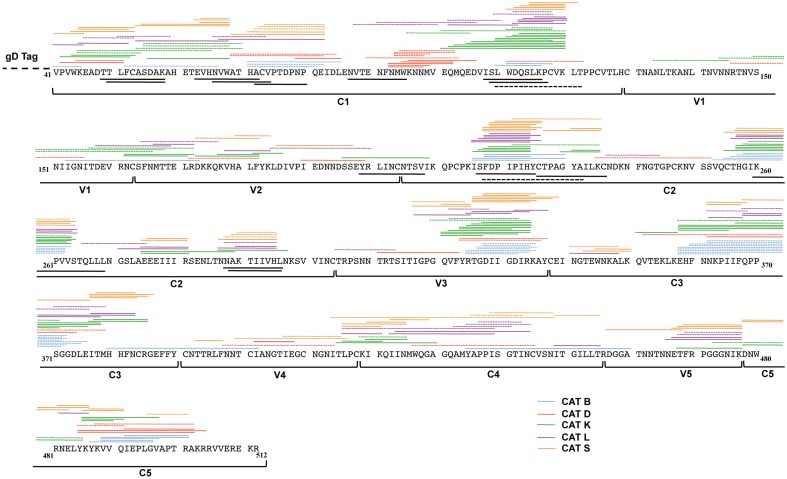
CAT cleavage maps of Env-A244. Env-A244 was subjected to CAT digestion for 90 min and 16 hrs. The peptide fragments were separated by UFLC, analyzed on an LCMS-IT-TOF mass spectrometer, and then identified using the MASCOT database. The numbering of Env-A244 is based on HXB2 numbering. Env-A244 also contains a gD tag at its N-terminus, the sequence of the gD tag is not shown. The different regions of Env-A244 (C1, V1, V2, C2, V3, C3, V4, C4, V5, and C5) are denoted under the sequence. The Env-A244 peptides generated from CAT B, (blue line) CAT D (red line), CAT K (green line), CAT L (purple line), and CAT S (orange) are shown The solid and the dotted lines above the sequence represent the peptide fragments identified at the 90 min and the 16 hrs time point, respectively. These fragments could contain potential MHC class I and class II epitopes. The solid black lines below the sequence represent MHC class I epitopes, while the dashed black lines represent MHC class II epitopes as reported in the Las Alamos database.

The peptides derived from CAT B, D, K, L, and S degradation of Env-A244 at 90 min (solid lines above the Env-A244 sequence) and additional peptides generated at 16 hrs (dotted lines above the Env-A244 sequence) were identified by mass spectrometry (Figure 3). The MHC class I and class II epitopes for Env-A244 as reported in the Los Alamos database are shown respectively beneath the amino acid sequence as a solid or hatched black line. Each of the cathepsin Env-A244 degradations generated clusters of potential peptide MHC class I and class II precursor epitopes (Figure 3 and Table 1) with dense clusters found in the C1, C2, V3, C3, C4, and C5 regions of Env-A244 that contained the CD4, chemokine, and majority of the neutralizing antibody binding sites. Each of the CAT generated varying number of peptides. The number of peptides produced was relatively consistent at 90 min and at 16 hrs with the exception of CAT B and D (Table 1). The concentration of individual peptides derived from each of the CAT degradations of Env-A244 was not assessed. Therefore, there could be varying concentrations of antigenic peptides in each of the CAT degradations.

**Table 1:** Env-A244 peptides generated by CAT and proteasomal cleavage of CAT degradation products.

Protease(s)	# of peptides identified^a^	Range of peptide length	Reported MHC class I epitopes^b^	Reported MHC class II epitopes^b^
CAT B 90 min	13	5–26	Yes	No
CAT B 16 hrs	37	5–19	Yes	No
CAT B+Proteasomes	37	5–19	Yes	No
CAT D 90 min	14	5–20	No	No
CAT D 16 hrs	26	5–28	Yes	No
CAT D+Proteasomes	24	5–27	Yes	No
CAT K 90 min	60	5–20	Yes	No
CAT K 16 hrs	78	5–23	Yes	Yes
CAT K+Proteasomes	68	5–24	Yes	No
CAT L 90 min	47	5–21	Yes	No
CAT L 16 hrs	48	5–24	Yes	No
CAT L+Proteasomes	71	5–26	Yes	No
CAT S 90 min	65	5–15	Yes	No
CAT S 16 hrs	56	5–17	Yes	No
CAT S+Proteasomes	51	5–15	Yes	Yes

a The number of Env-A244 peptides identified by mass spectrometry.

b As reported in the Los Alamos database.

**Figure 4:**
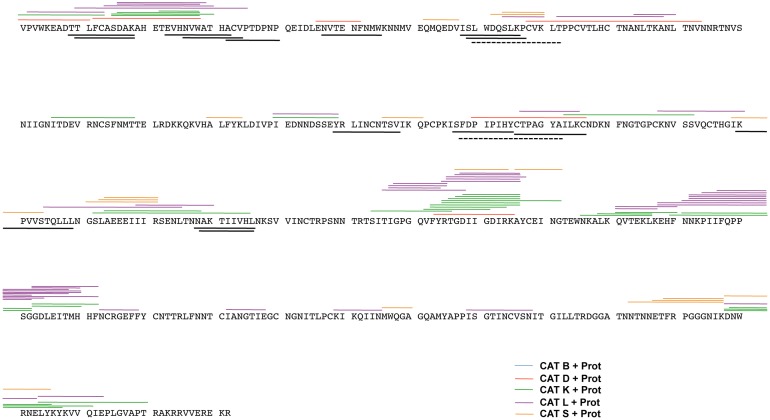
Proteasomal cleavage maps following CAT degradation of Env-A244. Env-A244 was subjected to CAT digestion for 90 min followed by proteasomal degradation for 16 hrs. The peptide fragments were separated by UFLC, analyzed on an LCMS-IT-TOF mass spectrometer, and then identified using the MASCOT database. The additional Env-A244 peptides generated from proteasomal degradation of CAT B (blue line), CAT D (red line), CAT K (green line), CAT L (purple line), and CAT S (orange) digestions are shown. The lines above the sequence represent additional peptide fragments identified after proteasomal degradation. These fragments could contain potential MHC class I and class II epitopes. The solid black lines below the sequence represent MHC class I epitopes, while the dashed black lines represent MHC class II epitopes as reported in the Las Alamos database.

Generally in a cell these CAT generated peptides can be retrotranslocated into the cytosol for further degradation by the proteasomes, therefore we incubated the cathepsin degradation products with purified proteasomes in vitro. When the 90 min CAT degradations were subjected to proteasomal degradation (Figure 4), in some cases, an alternative peptide repertoire was generated even though there was no apparent significant increase in the number of peptides identified by mass spectrometry with the exception of CAT L (Figure 4 and Table 1). The majority of the reported Env-A244 epitopes were present in the peptides generated by the cathepsin degradation (Table 2). Although proteasomal cleavage of the cathepsin degradation products destroyed approximately 50% of the peptides containing MHC class I epitopes, thus potentially removing the epitope for presentation through the alternative MHC class I pathway, it also generated 3 MHC class I epitopes that were previously absent in the peptides identified from CAT degradation (Table 2). The CAT generated peptides that contained known MHC class II epitopes were not affected by further treatment with proteasomes (Table 2).

**Table 2:** Identification of the reported epitopes following the proteolytic digest of Env-A244 sequence.

aEpitope	Proteasome	bCAT	cCAT+Prot
**MHC Class I**			
TTLFCASDAK	ABSENT	PRESENT	ABSENT
TLFCASDAK	ABSENT	PRESENT	PRESENT
EVHNVWATHA	ABSENT	ABSENT	PRESENT
NVWATHACV	ABSENT	ABSENT	PRESENT
ACVPTDPNP	ABSENT	PRESENT	PRESENT
NVTENFNMW	ABSENT	ABSENT	ABSENT
ISLWDQSLK	ABSENT	PRESENT	ABSENT
SLWDQSLKP	ABSENT	PRESENT	ABSENT
YRLINCNTSV	ABSENT	PRESENT	ABSENT
SFDPIPIHY	ABSENT	PRESENT	ABSENT
CTPAGYAILKC	ABSENT	ABSENT	PRESENT
KPVVSTQLLL	ABSENT	PRESENT	ABSENT
NAKTIIVHL	ABSENT	PRESENT	ABSENT
AKTIIVHL	ABSENT	PRESENT	ABSENT
**MHC Class II**			
LWDQSLKPCVKLT	ABSENT	PRESENT	PRESENT
FDPIPIHYCTPAGYA	ABSENT	ABSENT	ABSENT

a The epitopes reported on the Los Alamos database.

b, c The fragments that contain these epitopes are generated by cleavage of Env-A244 either by CAT or by proteasomal cleavage of the CAT digests.

### Characterization of identified Env peptides

**Figure 5:**
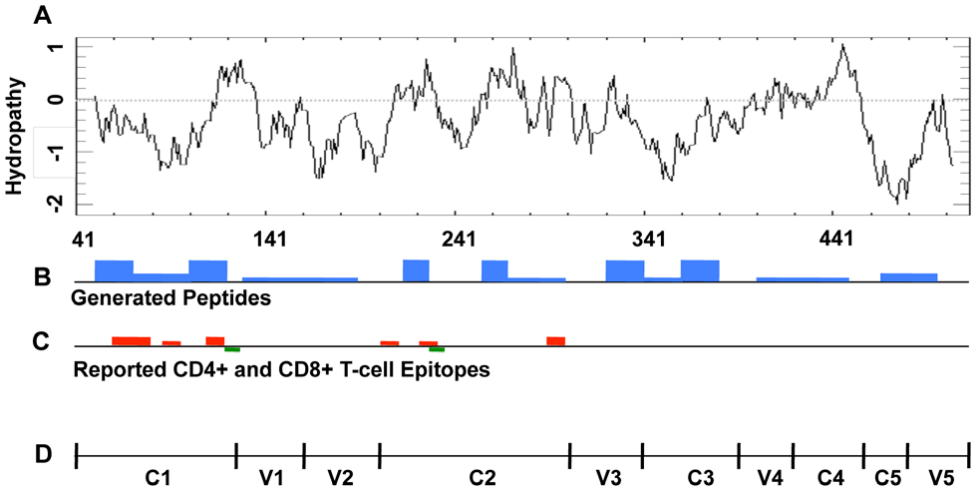
Kyte-Doolittle plot of HIV-1 Env-A244 in relation to peptide clusters. Hydrophobicity/hydrophilicity plots of HIV-1 Env-A244 (panel A) were generated with the Kyte-Doolittle algorithm (http://mobyle.pasteur.fr/cgi-bin/portal.py?form=pepwindowall). All the peptide fragments identified by mass spectrometry (Figure 3 A) are denoted by blue boxes (panel B). Red boxes represent the reported CD8^+^ T-cell epitopes and the green boxes represent the reported CD4^+^ T-cell epitopes (panel C). The constant and variable regions of Env-A244 are denoted in panel D. The greater the number of epitopes in each of the clusters, the greater is the height of the box.

The peptides generated from either the CAT degradation or the CAT followed by proteasomal degradation indicated that Env-A244 had regions of high and low epitope clusters. A Kyte-Doolittle hydrophobicity plot [37] was constructed for Env-A244 and although epitopes were generated throughout the length of the antigen, the high-density epitope clusters were observed in the hydrophobic regions of C2, V3, and C3 regions of Env-A244 (Figure 5). High-density epitope clusters were also observed in the C1 region of Env-A244, however these clusters were not in the highly hydrophobic region and epitope clusters were sparse in the hydrophilic region of the protein. In the Los Alamos database, few functional Env-A244 MHC class I and class II epitopes have been reported within the Env-A244 sequence. However, the majority of epitopes are clustered in the hydrophobic regions. Similar results were obtained with clade B gp140 protein (data not shown).

**Figure 6:**
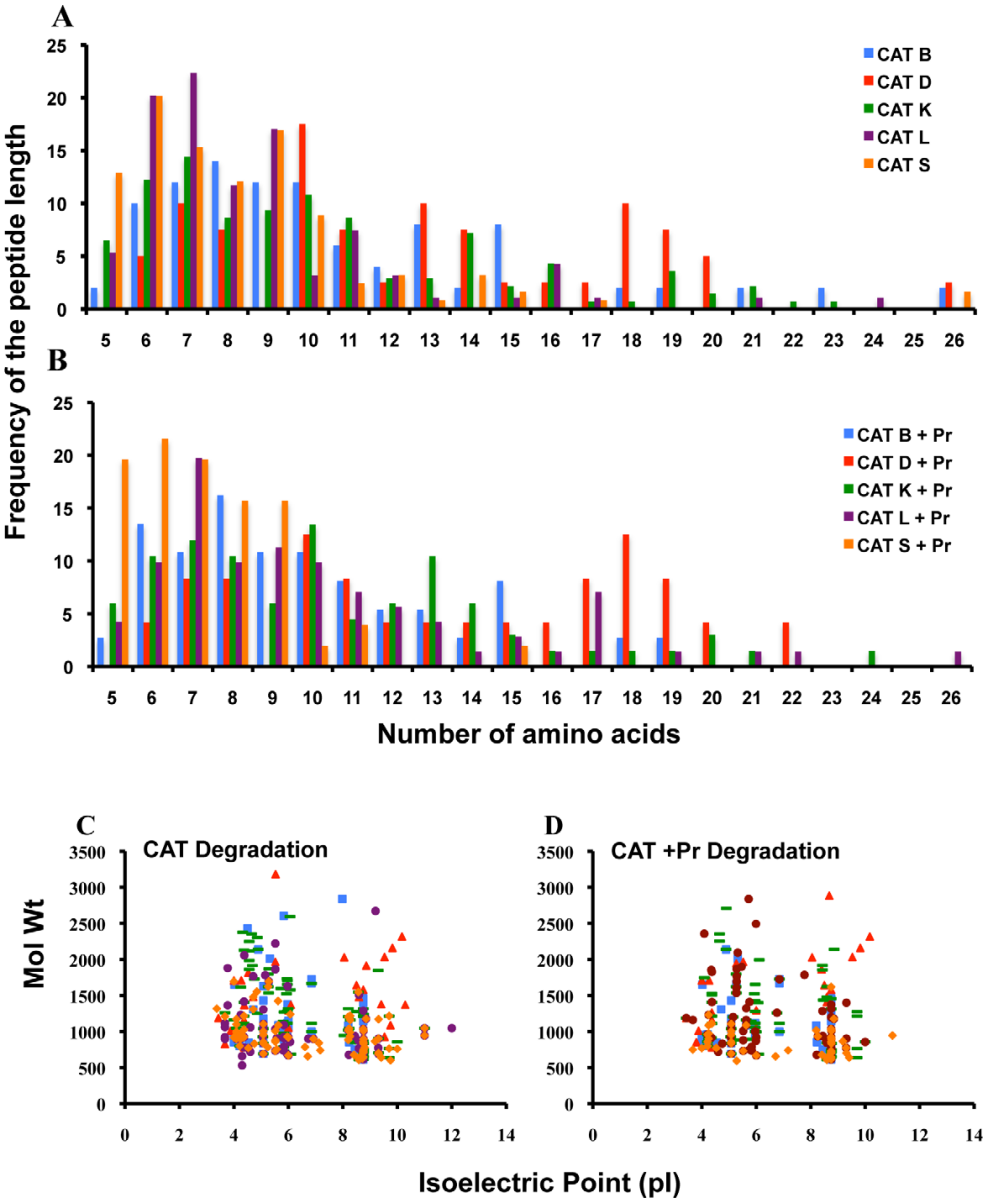
Biochemical characteristics of the CAT and CAT followed by proteasomal degradation products of Env-A244. The Env-A244 peptides that were identified in Figures 3 A and 3 B were plotted to compare the size and frequency of the peptides generated after individual CAT (panel A) or individual CAT followed by proteasomal degradation (panel B). The molecular weights (mol wt), and the isoelectric points (pI) of the peptides after each of the degradations were also analyzed (panels C, D). No significant differences in the amino acid length (panels A, B), pI or mol wt (panels C, D) of the peptides produced from the cleavage products of Env-A244 were observed.

The minimum required length for peptides to bind to MHC class I and class II molecules are 8 and 12 amino acids, respectively. CAT B, D, K, L, and S generated peptides ranging from 5 amino acids to greater than 19 amino acids with no significant differences in the length of peptides between the different degradations (Figures 6 A and 6 B). The majority of the peptides generated by CAT (Figure 6 A) or CAT followed by proteasomes (Figure 6 B) had the requisite peptide length that could potentially bind to MHC class I molecules. However, only about a third of the generated epitopes had the requisite peptide length required for binding to the MHC class II molecules. The peptides generated by the various CAT (Figure 6 C) or various CAT followed by proteasomes (Figure 6 D) clustered into two isoelectric ranges (pH 4–6 and pH 8–10) with no significant differences in the molecular weight of the peptides (http://au.expasy.org).

### Peptides derived from the CAT cleavage of Env-A244 stimulate IFN-γ from PMBC of RV144 immunized individuals

**Figure 7:**
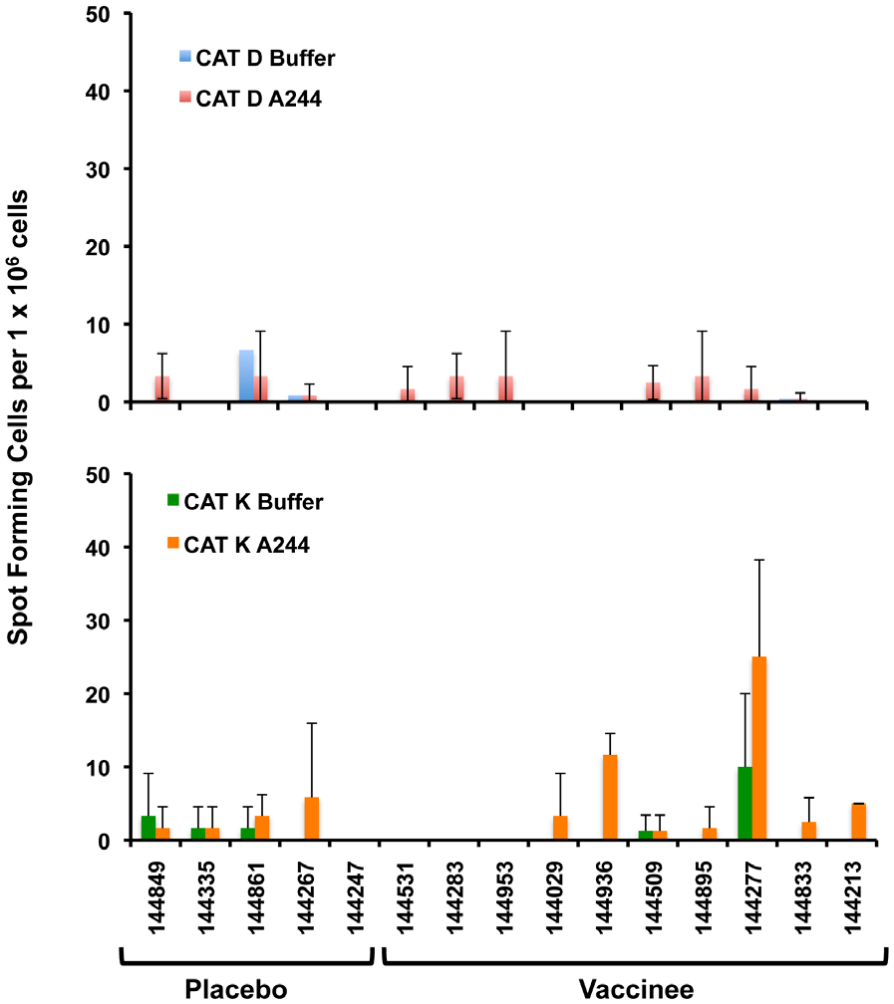
Env-A244 peptides derived by CAT K cleavage induce IFN-γ from the PMBC of RV144 volunteers. Peptides derived from the proteolytic cleavage of Env-A244 by CAT D (red bars) and K (orange bars), or the respective buffers (blue and green bars) used for the degradation were incubated with PBMC from five placebos and 10 vaccinees from the RV144 phase III trial and examined for the generation of IFN-γ by ELISPOT. Stimulation of PBMC with peptides generated from CAT D cleavage of Env-A244 did not generate increased levels of IFN-γ when compared to the placebos. Two of the vaccinees generated increased levels of IFN-γ following stimulation with peptides generated from CAT K cleavage of Env-A244 when compared to the placebos. The data represent the mean ± S.D. of triplicate wells.

**Figure 8:**
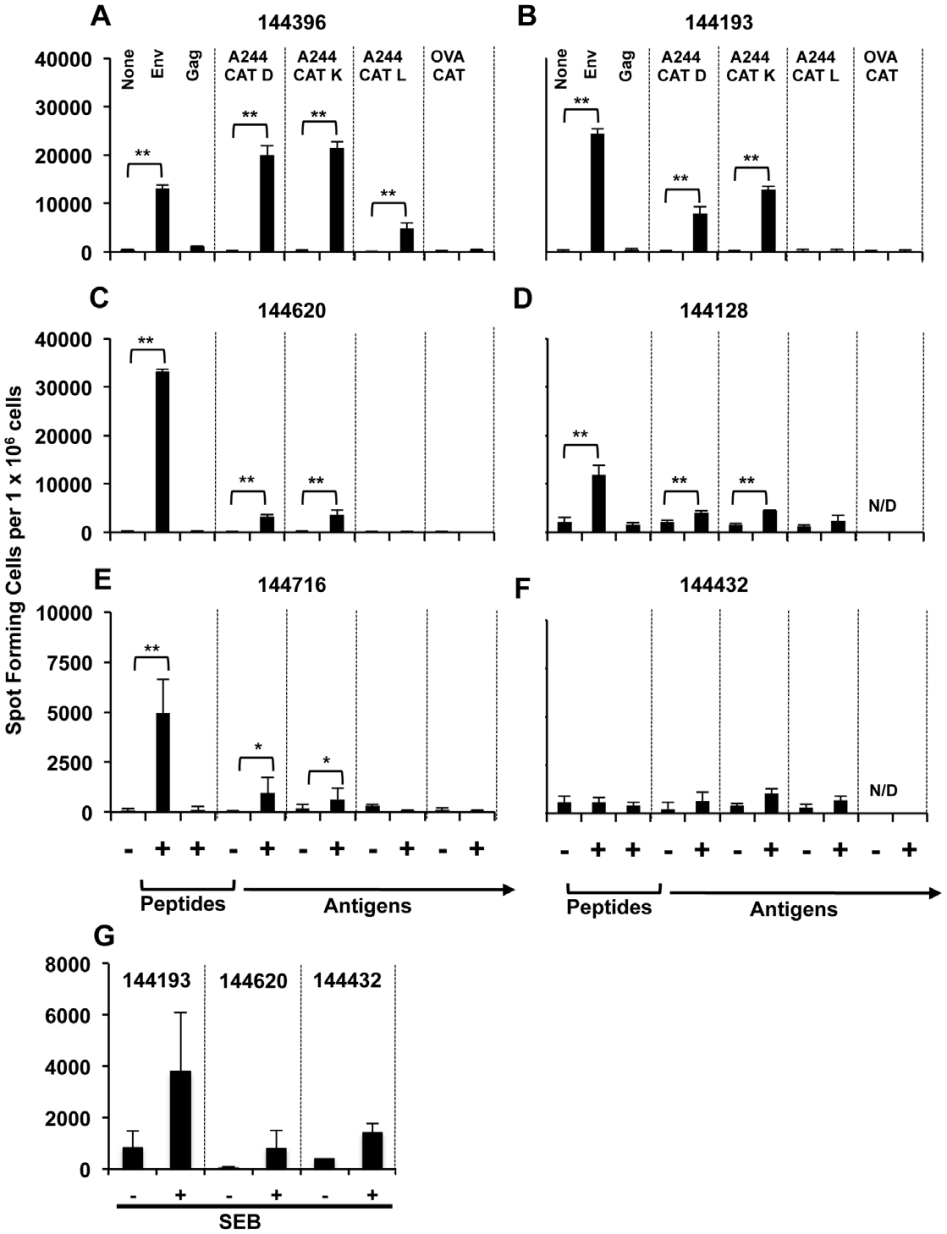
Env-A244 peptides derived by CAT D, K, and L cleavage induce IFN-γ from HIV-1 Env-specific CD4^+^ T-cells. CAT D, K, and L digests of Env-A244 were incubated with Env-specific CD4^+^ T-cell lines generated from six RV144 phase III trial volunteers. The Env-specific T cell lines were incubated with Env-CMDR peptide pool, Gag-CMDR peptide pool, media containing the buffers used for CAT degradation, and CAT D, K, and L degraded ovalbumin (OVA) as a non-HIV antigen control. The CD4+ T cell lines were then analyzed for the induction of IFN-γ by ELISPOT. No IFN-γ was detected in the CAT buffer controls or from the Gag-CMDR peptide pool (panels A–F); Four of the six cell lines tested with CAT degraded OVA also did not induce IFN-γ (panels A–C and E). Five out of the six cell lines (panels A–E) incubated with the Env-CMDR peptide pool induced IFN-γ. These same cell lines (A–E) also induced IFN-γ when stimulated with Env-A244 peptides derived from CAT D and K degradations. The cell line that did not induce IFN-γ with either Env-CMDR peptide pool or Env-A244 peptides derived from CAT D, K and L degradations, is a cell line derived from a placebo volunteer (panel F). Only one out of the 6 cell lines (panel A) incubated with Env-A244 peptides derived from CAT L induced IFN-γ. CD4^+^ T-cell lines derived from 144193 and 144620 (vaccinees) and 144432 (placebo) were tested for their response to SEB (panel G). All three of the cell lines showed a response to SEB demonstrating that the cells are functional. The data represent the mean ± S.D. of triplicate wells. *denotes p≤0.05 and **denotes p≤0.01. N/D: Not determined.

**Figure 9:**
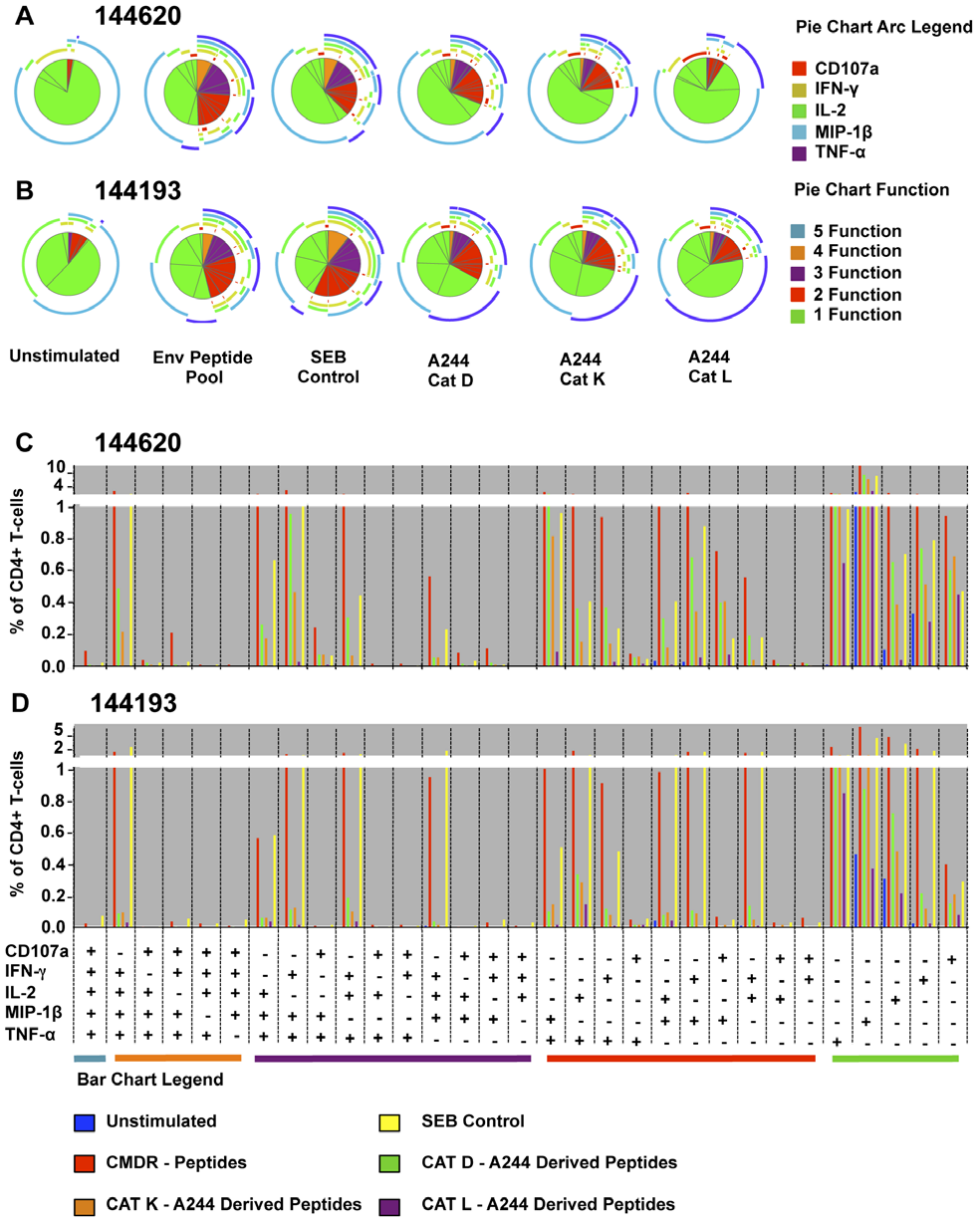
Env-A244 peptides derived by CAT D, K, and L cleavage induce a poly-functional cytokine response from antigen-specific CD4^+^ T-cell lines. HIV-1 Env specific CD4^+^ T cell lines derived from two volunteers from the RV144 phase III trial (144620 and 144193) were stimulated with either the CMDR Env peptides, SEB, peptides generated from Env-A244 cleaved by CAT D, CAT K, or CAT L, or cultured in media only. The CD4+ T cells were analyzed for the generation of CD107a (red), IFN-γ (brown), IL-2 (light green), MIP1β (light blue) and TNF-α (purple) by flow cytometry. The pie charts (panels A and B) demonstrate the fraction of cells with one (green), two (red), three (purple), four (orange), or five (light blue) cytokines generated from the same cell, and the circular lines arching the pie chart represent the five cytokines/chemokines examined. The data is denoted in a graphical format in panels C and D as a percentage of CD4^+^ T-cells capable of generating each of the 31 possible cytokine combinations. The color designations for each of the experimental conditions shown in panels C and D are as follows: unstimulated (blue bar); SEB (yellow bar); CMDR peptides (red bar); CAT-D Env-A244 derived peptides (green bar); CAT-K Env-A244 derived peptides (orange bar); and CAT-L Env-A244 derived peptides (purple bar).

The functional relevance of peptides derived from degradation of Env-A244 by CAT D, K, and L were evaluated using either peripheral blood mononuclear cells (PBMCs) obtained from RV144 volunteers (Figure 7) or from CD4^+^ T-cell lines derived from RV144 volunteers (Figures 8 and 9). The number of IFN-γ producing cells was determined by an ELISPOT assay, using PBMCs from the peak of immunogenicity at the 2-week time point following the completion of the RV144 immunization schedule. The Env-A244 peptide repertoire derived from degradation by CAT D and K were examined individually for their potential to induce IFN-γ. Although the ELISPOT responses were weak, the peptides generated from CAT K induced IFN-γ in 2 out of the 10 vaccinees tested (Figure 7, bottom panel) that were at least twice the background (buffer control). The ELISPOT well images from the two positive responders stimulated with peptides derived from Env-A244 CAT D and CAT K digests including images of wells stimulated with media, PHA, CMV peptide pool, and CAT buffers are shown in Figures S1 and S2. Although the ELISPOT responses are low, the results are consistent with a recent study analyzing the Env-specific IFN-γresponses generated from PBMCs of RV144 volunteers. Furthermore, depletion studies indicated that the IFN-γgeneration was from CD4+ T cells [38].

The peptides generated from CAT D were ineffective in stimulating IFN-γ (Figure 7). This could either be due to the low frequency of Env-specific CD4^+^ T-cells in the PBMC of the vaccinees or insufficient concentrations of the relevant peptides generated from the CAT cleavage of Env-A244.

Six different CRF01_AE Env-specific CD4^+^ T-cell lines derived from RV144 volunteers were used to evaluate the Env-A244 CAT-derived peptides by ELISPOT (Figure 8). The CAT D and K derived Env-A244 peptides stimulated IFN-γ production in five out of the six cell lines tested (Figure 8, panels A–E) while the CAT L derived peptides stimulated IFN-γ in only one out of the six cell lines (Figure 8 A). The responses in all cases were significantly higher than the appropriate buffer controls (Figure 8, panels A–E). To ensure that the IFN-γ response to the CAT-derived peptides was specific to Env A244, a non HIV-1 protein (ovalbumin, OVA) was degraded individually with CAT D, K, and L and the degradations were combined. The OVA-derived peptides did not induce IFN-γ (Figure 8, panels A–C and panel E). Five out of the six Env-specific T-cell lines induced IFN-γ in response to pooled CMDR Env peptides, and none of the six cell lines induced IFN-γ in response to pooled CMDR Gag peptides thereby confirming Env specificity (Figure 8, panels A–E). One (144432, placebo) of the 6 cell lines did not react to either the CMDR Env peptide pools or to the CAT degradation products (Figure 8 F). CD4^+^ T- cell lines derived from 144193 and 144620 (vaccinees) and 144432 (placebo) were tested for their response to SEB (Figure 8 G). All three of the cell lines showed a response to SEB demonstrating that the cells are functional.

The epitope specificity of the six cell lines was determined using 15-mer overlapping CMDR peptides derived from CM235-Env sequence, and compared to the CAT D, K, and L-derived Env A244 peptides. The sequences that matched/aligned to 15-mer overlapping CMDR peptides that were specific to each of the five T cell lines is shown in Table 3. Additional CMDR peptides stimulated the T cell lines but were absent in the Env-A244 CAT-derived peptides and are therefore not shown in Table 3. Of the 5 cell lines that induced IFN-γ, cell lines 144396 and 144716 were stimulated predominantly from peptides derived from the constant regions of Env-A244, whereas 144193 and 144620 were stimulated predominantly from peptides derived from the constant, V1, and V2 regions of Env-A244 and one cell line (144128) was specific to a peptide in the V3 region.

**Table 3:** Comparison of the sequences of the peptides derived from Env-CMDR to the peptides generated from the CAT degradation of Env-A244 that induced IFN-γ as measured by ELISPOT analysis.

ELISPOT data	CAT D	CAT K	CAT L
**144396 (** **Figure 7A** **)**			
DPNPQEIHLENVTENF^a^	ATHACVPT**DPNPQEID** b		
TENFNMWKNNMVEQM	**NMWKNNMVEQ**		V**TENFNMWKNNMV**
	**NFNMWKNNMVEQM**QED		**NFNMWKNNMVEQM**QED
AEEEIIIRSENLTNNA		**EEIIIRSENLTNNA**	GSL**AEEEIIIR**
TQVTEKLKEHFNNKTI		**EHFNNKPI**IFQPPS	
		**EHFNNKPI**IFQPPSGGDLE	
		**EHFNNKPI**IFQPPSGGDLEIT	
AMYAPPISGRINCVSNI	WQGAGQ**AMYAPPISGTINC**		LPCKIKQIINMWQGAGQ**AMYAPPI**
			**AMYAPPISG**
			INMWQGAGQ**AMYAPPISGTIN**
RPGGGNIKDNWRSELY		**GGNIKDNWRNELY**	
**144193 (** **Figure 7B** **)**			
DVISLWDQSLKPCVKL	**WDQSLKPCVKL**TPPCVTL	EQMQE**DVISLWDQSLKPCVK**	
		QMQE**DVISLWDQSLKPCVK**	
		QE**DVISLWDQSLKPCVK**	
		E**DVISLWDQSLKPCVK**	
		**DVISLWDQSLKPCVK**	
		**LWDQSLKPC**	
		**LWDQSLKPCVK**	
VHALFYKLDIVPIEDNK	RDKKQK**VHALF**	DKKQK**VHALF**	
		K**VHALF**	
		**VHALFYK**	
		**FYKLD**	
FNGTGPCKNVSSVQCTH	KCNDKN**FNGTGPCKNVSS**		
**144620 (** **Figure 7C** **)**			
MWKNNMVEQMQEDVISL	ENFN**MWKNNMVEQM**		
DVISLWDQSLKPCVKL	**WDQSLKPCVKL**TPPCVTL	EQMQE**DVISLWDQSLKPCVK**	
		QMQE**DVISLWDQSLKPCVK**	
		QE**DVISLWDQSLKPCVK**	
		E**DVISLWDQSLKPCVK**	
		**LWDQSLKPCVK**	
ITSVSNTIGNITDEVR		NN**RTNVSNIIGNITDEVR**NCSFN	
		**SNIIGNITDEVR**NCSFNMT	
TTELRDKKQKVHALFY	**RDKKQKVHALF**	**DKKQKVHALF**	
VHALFYKLDIVPIEDNK	**RDKKQKVHALF**		
KLKEHFNNKTIIFQPPS	VTE**KLKEHFNNKPIIFQPPS**GGDLEITM	**EHFNNKPIIFQPPS**	
		**EHFNNKPIIFQPPS**GGDLE	
		**EHFNNKPIIFQPPS**GGDLEIT	
		**NNKPIIFQPPS**GGDLE	
FNNKTIIFQPPSGGDL	VTEKLKEH**FNNKPIIFQPPSGGDL**EITM		
INMWQGAGQAMYAPPI	**WQGAGQAMYAPPI**SGTINC		
IKDNWRSELYKYKVV		GGN**IKDNWRNELY**	
WRSELYKYKVVQIEPL	**YKYKVVQIEPL**GVAPTRA	**KYKVVQIEPLG**	
	**YKYKVVQIEPL**GVAPTRAK		
	**YKYKVVQIEPL**GVAPTRAKR		
	**KVVQIEPLG**VAPTRA		
YKYKVVQIEPLGIAPT	**YKYKVVQIEPL**GVAPTRA		
	**YKYKVVQIEPL**GVAPTRAK		
	**YKYKVVQIEPL**GVAPTRAKR		
	**KVVQIEPLG**VAPTRA		
VQIEPLGIAPTRAKRR	**YKYKVVQIEPL**GVAPTRA		
	**YKYKVVQIEPL**GVAPTRAK		
	**YKYKVVQIEPL**GVAPTRAKR		
	**KVVQIEPLG**VAPTRA		
**144128 (** **Figure 7D** **)**			
TRPSNNTRTSIPIGPG	TRTSITIGPGQVF	**TRTSITIGPG**QVF	
**144716 (** **Figure 7E** **)**			
FNNKTIIFQPPSGGDL	VTEKLKEH**FNNKPIIFQPPSGGDL**EITM	EH**FNNKPIIFQPPS**	
	**NKPIIFQPPSGGD**	EH**FNNKPIIFQPPSGGDL**E	
		EH**FNNKPIIFQPPSGGDL**EIT	
		**NNKPIIFQPPSGGDL**E	
IIFQPPSGGDLEITMH	VTEKLKEHFNNKP**IIFQPPSGGDLEITM**	EHFNNKP**IIFQPPS**	
	NKP**IIFQPPSGGD**	EHFNNKP**IIFQPPSGGDLE**	
		EHFNNKP**IIFQPPSGGDLEIT**	
		NNKP**IIFQPPSGGDLE**	
INMWQGAGQAMYAPPI	**WQGAGQAMYAPPI**SGTINC		
**114432 (** **Figure 7F** **)**			
ND	ND	ND	ND

a Env-CMDR peptide sequences that induced IFN-γ from T-cell lines generated from the RV144 phase III trial.

b Peptides derived from the cathepsin cleavage of Env-A244. The amino acids in bold represent sequences of amino acids found in both CMDR and CAT-derived peptides.

Two (144193, 144620) of the Env-specific CD4^+^ T-cell lines that responded to peptides derived from Env-A244 CAT degradations (Figures 8 B and 8 C) were analyzed by flow cytometry for intracellular cytokine production. (Figure 9 A–D). Stimulation of these cell lines individually with CAT D, K, and L-derived peptides induced multiple inflammatory cytokines as measured by intracellular cytokine staining (ICS), demonstrating the poly-functional response of the cells (Figure 9 A–D). The CAT D, K, and L-derived peptides from Env-A244 induced up to three cytokines from the same cell, and a small population of cells stimulated with the CAT D and K-derived peptides induced four cytokines from the same cell.

## Discussion

Antigen processing is highly complex involving a number of different components some of which overlap between MHC class I and class II pathways. In this study, we examined the ability of purified proteasomes from activated CD4^+^ T-cells and commercially available CAT to proteolytically cleave HIV-1 Env-A244, one of the vaccine components of the RV144 phase III trial [35]. Although in vivo, the degradation patterns may differ from our in vitro studies, nonetheless it sheds light on the influence of proteasomes and CAT on the generation of peptides containing potential MHC class I and class II epitopes.

The preliminary immunological data from the RV144 trial indicated that both the cellular and humoral immune responses were biased towards the Env antigen with a dominant CD4^+^ T-cell response [38]. Our results indicate that Env-A244 was resistant to proteasomal cleavage but susceptible to proteolytic degradation by CAT and to proteasomes following CAT degradation. Moreover, components of these CAT/proteasome digests were recognized in bulk PBMC culture ELISPOT and confirmed in epitope specific CD4^+^ T-cell lines derived from RV144 vaccinees.

It has been demonstrated that RNaseB, a folded and glycosylated protein is susceptible to proteasomal cleavage only if the protein folding can be prevented by reduction and alkylation [28]. Env-A244 is also a folded protein and highly glycosylated with mannose residues, which could interfere with the ability of the proteasomes to cleave the protein. Interestingly, following either removal of glycans of both the high-mannose and the complex type linked through asparagine to the protein, or after reduction and alkylation, Env-A244 remained resistant to proteasomal cleavage. The reason for this is currently unknown. However, proteasomal resistance was not dependent upon the glycosylation since both CHO-expressed Env-A244 gp120 and H9 cell-expressed gp140 could not be cleaved by proteasomes even though the composition of the sugars would be expected to be different in the two proteins [39].

We analyzed and identified the peptides produced after CAT degradation or CAT followed by proteasomal degradation by mass spectrometry. The identified peptides ranged from 5 to 25 amino acids in length and some of these contained MHC class I and class II epitopes as reported in the Los Alamos database. The dense clusters of peptides from the CAT D, K, and L cleavage of Env-A244 were found in the C1, C2, C3, C4, C5, and the V3 region of the gp120 antigen and these regions displayed domains that were both hydrophobic and hydrophilic, but were predominantly hydrophobic in comparison to the V1, V2, V4, and V5 regions that were dominated by hydrophilic regions. The location of eight protease cleavage sites on HIV-1 gp120 recognized by CAT D, L, and S and the effect of CAT cleavage on the binding of gp120 to CD4-IgG and neutralizing antibodies has been reported [40].

The endo-lysosomal compartment contains multiple cathepsins that are differentially expressed based on the activation status of the different antigen presenting cells [18]. Macrophages and activated antigen presenting cells have high concentrations of cathepsins and rapidly degrade antigens. In contrast, dendritic cells and non-activated cells contain low concentrations of cathepsins and thus preserve antigenic epitopes. Adjuvants such as liposomes containing lipid A and poly I:C are capable of activating TLR-4 and TLR-3, respectively [41], [42] by either increasing the enzymatic activity or by increasing the cathepsin mRNA expression. Adjuvants could also have a direct/indirect effect on functional activity of the cathepsins. Aluminum salts have been shown to inhibit the proteolytic activity of cathepsin D and could influence the generation of epitopes [43], [44]. However, as multiple cathepsins are present the in the endo-lysosomal compartment, these epitopes can still be generated and presented.

CAT B generated peptides that were too large in size to directly bind to either MHC class I or MHC class II molecules without further processing. Although CAT S generated Env-A244 peptides ranging in length from 5–17 amino acids (Table 1) the majority of peptides were too small to bind to MHC class II molecules as determined by the frequency of the peptide length (Figure 5). The number of CAT fragments that could potentially give rise to MHC class I or class II epitopes were 148 (60.5%) compared to 97 (39.5%) from the hydrophilic regions and these numbers did not significantly alter after proteasomal degradation of the CAT-derived peptides (Figure 3 and 4, and Table 1). Interestingly for the correct protein folding, hydrophobic regions are important and this may lead to these regions being highly conserved and thus influencing the immunogenicity [45].

The functionality of the peptides containing potential MHC class II epitopes was demonstrated using PBMCs from subjects enrolled in the RV144 phase III trial (5 placebos and 10 vaccinees) and T-cell lines (predominantly comprised of CD4^+^ T-cells) derived from PBMC of six different RV144 volunteers. Our experiments demonstrated that peptides generated from the proteolytic cleavage of Env-A244 by CAT K and not CAT D induced the secretion of IFN-γ in 2 out of the 10 vaccinees tested. None of the placebos induced IFN-γ In the CAT K degradation, 78 peptides were identified by mass spectrometry (Table 1) containing potential and reported MHC class I and class II epitopes, while 26 peptides were identified in the CAT D degradation that contained no reported MHC class II epitopes. One possible explanation for the absence of IFN-γ secretion by peptides-derived from CAT D degradation, could be due to a low frequency of peptide-specific T cells in the PBMCs. However when we used antigen-specific CD4+ T-cell lines generated from RV144 vaccinees, IFN-γ positive cells were induced by peptides derived from CAT D, K, and L degradation of Env-A244. The differences in the reactivities between the different CAT degradation products within one T cell line and the variations between the 6 T-cell lines could be due to differences in the relative amount of peptide fragments present within each of the CAT degradation products and the respective HLA backgrounds of the vaccinees from whom the cell lines were derived. In our assay, the peptides are generated from CAT cleavage and therefore the concentration of the generated peptides is not uniform and controlled as would be the case in a traditional antigen presentation assay wherein a fixed concentration of individual synthetic peptides is used. The CAT-derived peptides also induced a polyfunctional T cell response.

Peptide fragments derived from CAT D and K containing the sequence DKKQKVHALF (Table 3) induced IFN-γ from CD4^+^ T-cell lines obtained from two different vaccinees (144620, and 144193). This sequence is contained in the V2 loop of gp120. Recently, it has been demonstrated that antibodies specific to a fusion protein containing the V1 and V2 regions of gp120 (gp70-V1V2) correlated inversely with the risk of HIV-1 infection in the RV144 phase III trial and preliminary epitope mapping has identified this region (in/around aa 164–178 of V2) as the site of both polyclonal and monoclonal binding [36], [46], [47]. Interestingly, the V2 epitope identified in proteolytic digests is also recognized by T cells. Our studies show that the sequence DKKQKVHALF is produced by degradation of Env-A244 by CAT D and K. This sequence induced a polyfunctional cytokine response including the generation of IFN-γ and Th2 like cytokines from CD4^+^ T-cell lines derived from RV144 vaccinees, thus underscoring the importance of CD4^+^ T-cells in the generation of antibodies. The production of these cytokines was also detected from bulk PBMC cultures stimulated with linear peptides from 92TH023 (one of the vaccine immunogens) and appeared to trend toward lowered risk of HIV infection in the RV144 trial [36].

Our studies highlight the importance of understanding the characteristics of the antigen, the route, adjuvant, and the vehicle of delivery, its influence on antigen processing, and the various proteases required, which could affect the induction of an immune response towards either a humoral or a cellular response. Generally, HIV-1 Env antigens induce a poor CD8 T cell response compared to the depth and breadth seen with Gag, which may be the result of differential processing of HIV-1 antigens [48].

**Figure 10:**
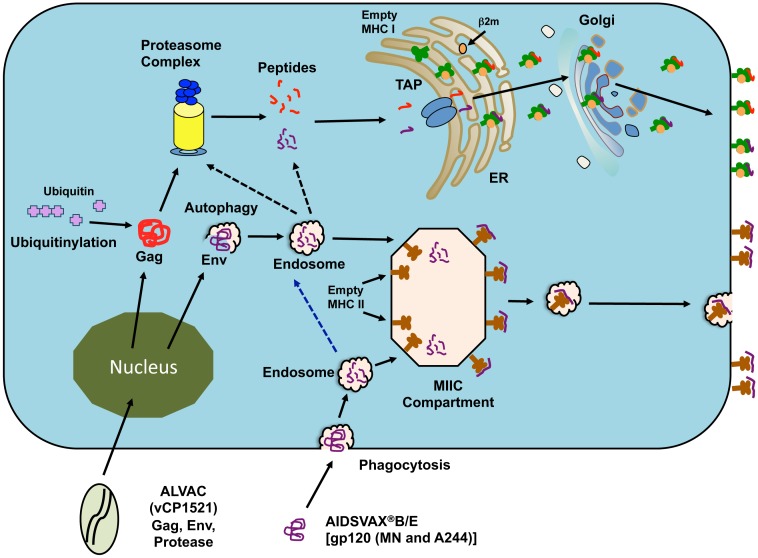
A schematic representation of the differential processing of the components of the RV144 vaccine. The RV144 vaccine consists of canarypox vector ALVAC (vCP1521), carrying HIV clade B and circulating recombinant form (CRF)01_AE *gag*, *pro* and *env* genes, and AIDSVAX®B/E genetically engineered gp120 protein from viruses of subtypes B and CRF01_AE. After entry into the cell, the ALVAC canary pox vector will generate protein antigens in the cytosol after transcription and translation. These proteins are then processed through the classical MHC class I pathway involving the proteasomal complex. The phagocytosed AIDSVAX®B/E protein antigens will enter the endosome/lysosome compartment and will be cleaved by the CAT. Some of the peptides generated within the endosome/lysosome compartment can be retrotranslocated into the cytosol and enter the classical MHC class I pathway. The peptides containing the MHC class II epiotpes enter the MIIC compartment from the endosome/lysosome where they are then loaded onto class II molecules and transported to the cell surface.

The RV144 phase III trial vaccine regimen consisted of four doses of a recombinant canary pox vector-based priming immunogen, ALVAC (vCP1521) administered at 0, 4, 12, and 24 weeks, and two doses of AIDSVAX®B/E co-administered at 12 and 24 weeks. AIDSVAX®B/E consists of genetically engineered HIV-1 gp120 proteins from viruses of subtypes B and CRF01_AE. We hypothesize that each component of the vaccine might be processed and presented in a different manner, and this is schematically represented in Figure 10. Theoretically, the ALVAC canary pox vector will generate protein antigens in the cytosol after transcription and translation, while the AIDSVAX®B/E protein antigens will be phagocytosed into endosome/lysosome compartment. Normally, Gag is proteolytically cleaved by the proteasome and follows the classical MHC class I pathway. Since HIV-1 Env was resistant to proteasomal degradation (Figure 1 A and Table 2) we hypothesize that the Env within the cytosol could potentially be processed by a mechanism known as autophagy (phagocytosis within the cell) wherein antigens are taken up into endosomes and cleaved by CAT. These peptides can either be directly loaded onto MHC class II molecules or retrotranslocated into the cytosol where they can then enter the proteasome/classical MHC class I pathway. Our results show that the potential Env-A244 MHC class I epitopes are destroyed to a greater extent as a result of proteasomal cleavage following CAT digestion (Table 2). This could limit the relative amounts of peptides available for presentation by the classical MHC class I pathway and may be a further possible explanation for the poor Env-specific CD8^+^ T-cell response seen in the RV144 Phase III trial. Although, other studies have demonstrated a strong and broad Gag-specific CD8^+^ T-cell response [48]–[50] the weak Gag response seen in the RV144 phase III trial is perplexing.

The phagocytosed AIDSVAX®B/E protein is cleaved by CAT in the endosomes/lysosomes generating a peptide array containing MHC class I and MHC class II epitopes. These peptides can be retrotranslocated and can enter the MHC class I pathway. The peptides containing the MHC class II epiotpes enter the MIIC compartment where they are loaded onto class II molecules and then transported to the cell surface [19], [51]. In conclusion, our results demonstrate that Env-A244 is resistant to proteasomes but susceptible to CAT cleavage and the resulting peptides induced a polyfunctional cytokine response including the generation of IFN-γ from CD4^+^ T-cell lines derived from RV144 vaccinees. We have also identified a sequence in the V2 loop of gp120 that has recently gained importance as a potential antibody-binding site. This sequence was generated by CAT cleavage of EnvA244 and induced IFN-γ from CD4^+^ T-cell lines.

## Materials and Methods

### Ethics statement, protocol authorization, and regulatory approval

RV144 (WRAIR Protocol #900): This clinical trial [35] protocol and all related documents were approved by the following independent Institutional Review Boards (IRBs): Division of Human Subject Protection, Walter Reed Army Institute of Research; Ethical Review Committee for Research in Human Subjects, Ministry of Public Health, Thailand. (http://clinicaltrials.gov/ct2/show/NCT00223080?term=RV144&rank=2NCT00223080). RV229B (WRAIR Protocol #1386): This protocol “Apheresis of blood components from healthy volunteers for in vitro research” and all related documents were approved by the following independent Institutional Review Boards (IRBs): Division of Human Subject Protection, Walter Reed Army Institute of Research; Ethical Review Committee for Research in Human Subjects. All volunteers provided written informed consent following discussion and counseling by the clinical study team prior to enrollment and before any trial related procedures were performed.

### Cells

PBMCs from healthy volunteers were collected under a Walter Reed Army Institute of Research Institutional Review Board (IRB) approved protocol, RV229B. Previously described methods for the generation of activated CD4^+^ T-cells and assessment of their purity were used [10]. These cells were used for the purification of proteasomes [10]. To analyze the functional reactivity (ICS, ELISPOT) of the CAT generated peptides, PBMCs from the RV144 phase III trial were collected under an IRB approved protocol RV144.

### Reagents

CAT B, D, H, K, L, and S were commercially purchased from ENZO life sciences (Farmingdale, NY, USA). CHO-expressed Env-A244 (gp120) was a gift from GSID. Env-gp140 purified from H9 infected T-cells was purchased from ABL Inc., (Rockville, MD, USA). *E. coli* expressed Gag-p24 was obtained through a collaboration with The Catholic University of America. RNaseB and SEB were purchased from Sigma-Aldrich (St Louis, MO, USA). A peptide set matching the Gag (CM240) and Env (CM235) insert sequences in the MVA-CMDR vaccine vector was manufactured by JPT Peptide Technologies Inc. (Berlin, Germany). The Gag and Env peptide sets consisted of 95 and 138 individual peptides, respectively, of 15 to 18 amino acids in length overlapping by 10 to 12 amino acids and spanning each protein. Each peptide set was pooled by co-lyophilization at JPT Peptide Technologies Inc., to make complete CMDR-Gag and CMDR-Env peptide pools [52]. The CMDR-Env peptide pool is derived from the Env-CM235 sequence that is 92% homologous to the Env-A244 sequence.

### Expansion of Env-specific CD4+ T cells from RV144 vaccinees

The CD4^+^ T-cell lines were established as previously described [53]. Briefly, PBMC from RV144 vaccine volunteers were thawed, re-suspended at 1×10^7^ cells/ml and pulsed with CRF01_AE strain A244 gp120 (25 µg/ml) for 4 hrs at 37°C in complete medium (CM) composed of RPMI 1640 (Quality Biological Inc. Gaithersburg, MD, USA) supplemented with L-glutamine 4 mM, penicillin 100 U/ml, streptomycin 100 µg/ml and 10% heat-inactivated NHS (Gemini, CA. USA). Cells were diluted in CM, plated at 2×10^6^ cells/ml in 24-well plates (Costar, Lowell, MA, USA) and incubated at 37°C, 5% CO_2_. After 4 days the cells were fed with 10 U/ml recombinant IL-2 (rIL-2) (Boehringer-Mannheim, Germany). After one week of culture, and every 2–3 days thereafter, half of the medium was replaced with fresh medium with rIL-2 and split according to growth. After 15 days, the expanded T cells were re-stimulated non-specifically for another two weeks by using purified anti-human-CD3 antibody (BD Pharmingen, San Diego, CA, USA) bound to a 6-well plate, anti-human CD28 purified antibody (BD Pharmingen) in suspension, and a pool of mismatched irradiated PBMC from healthy donors. Two days after the re-stimulation, the cultures were enriched with 10 IU of rIL-2 (Roche, Indianapolis, IN, USA). Every 2 days, fresh 10% NHS -RPMI with or without rIL-2 was added to the culture as needed. Cultures were kept at 37°C, 5% CO_2_, and 95% humidity. Expanded CD4^+^ T-cells were cryopreserved until ready to test in an Intracellular Cytokine Staining (ICS) assay or IFN-γ ELISPOT.

### Deglycosylation and enzymatic degradation of Env-A244 and RNaseB

CHO-expressed Env-A244 (50 µg) and RNaseB (50 µg) were treated with Endo-F and Endo-H (Sigma-Aldrich) according to the manufacturer's instructions. The protein was then examined by SDS-PAGE to ensure that the protein was deglycosylated and then subjected to proteasomal degradation.

Based on previously described methods [10], [11], [40] 25 µg or 50 µg of Env-A244 were incubated with purified proteasomes (5 µg) or with each of the respective CAT (0.5 µg) for 16 hrs at 37°C unless otherwise stated. To ensure the specificity of proteasomes, epoxomicin was used as an inhibitor. The inhibitor was pre-incubated with the proteasomes before the addition of the antigen. The reactions were stopped by freezing the samples at −80°C. The proteasomal and CAT degradation products were analyzed on an LCMS-IT-TOF mass-spectrometry. A separate aliquot of the degradation products was analyzed by SDS-PAGE and stained with GelCode Reagent Blue Stain. *E. coli* expressed Gag-p24 (50 µg) and CAT degradation products of Env-A244 (50 µg) at the 90-min time point were proteolytically cleaved by purified proteasomes (5 µg) for 16 hrs and analyzed as mentioned above.

### Reduction and alklyation of Env-A244 and RNaseB

Env-A244 and RNaseB (150 µg each) were each diluted in 20 mM TRIS, pH 7.2 containing 20 mM DTT (Sigma-Aldrich) and heated for 30 min at 56°C. The reduced proteins were alkylated by the addition of 50 mg/ml of iodoacetamide (Sigma-Aldrich) in 20 mM TRIS buffer and incubated at room temperature for 30 min. The fractions were washed with 20 mM TRIS and concentrated, using an Amicon ultra-15 centrifugal filter device (10 kDa cut off, Millipore, Billerica, MA, USA) 800 g at 4°C. The alkylated proteins are denoted as A244-IAA and RNaseB-IAA.

### Separation and analysis of peptides

The CAT and proteasomal degradation products of Env-A244 were analyzed by LCMS-IT-TOF mass spectrometry by MS and tandem MS/MS as previously described [10]. Each sample was analyzed in duplicate. Peptides were identified using the Mascot Software (Matrix Science, London, UK) with the MS/MS ion search. The peptide MS tolerance was set to 0.2 Da and the MS/MS tolerance was set to 0.1 Da using the monoisotopic peaks. The searches were conducted using the known sequence of Env-A244 and the Swiss-Prot database.

### ELISPOT assay (PBMCS)

Briefly, 96-well flat bottom hydrophobic polyvinylidene difluoride membrane plates (Millipore) were coated over night at 4°C with anti-human IFN-γ monoclonal antibody (final concentration 5 µg/ml; Mabtech, Mariemont, OH, USA). PBMC from RV144 vaccines and placebos were re-suspended in RPMI supplemented with 10% normal human serum, 2 mM L-Glutamine (Invitrogen, NY, USA), 50 µg/ml streptomycin and 50 U/ml penicillin (Invitrogen) and plated at a concentration of 2×10^5^ cells/well. Wells containing PBMC and buffers used in CAT D or K degradations served as negative controls. Phytohemagglutinin (PHA; Sigma-Aldrich) and CMVpp65 peptide pool (JPT) were used as positive controls. PBMC plus CAT D or K degraded Env-A244 peptides at a final concentration of 20 µg/ml were tested in triplicate. Negative controls were performed in quadruplicate. After incubation at 37°C in 5% CO_2_ for 20 to 24 hrs, PBMC were removed by washing with PBS/0.05% Tween-20 (Sigma-Aldrich). Captured IFN-γ was detected by incubation for 2 hrs at 37°C with biotinylated anti-human IFN-γ monoclonal antibody (Mabtech) at 2 µg/ml in PBS/0.5% BSA. Following incubation, plates were washed with PBS/0.05% Tween-20 and avidin horseradish peroxidase complex was added for 1 hr at room temperature. Unbound complex was removed by washing 6 times with PBS and peroxidase staining was performed using AEC substrate (AEC substrate Kit, Vector Labs) according to the manufacturer's instructions. Plates were scanned with a C.T.L. S5 Core Analyzer (Shaker Heights, OH, USA) using ImmunoCapture software version 6.2. Spots were counted and subjected to quality control using ImmunoSpot software version 5. A positive IFN-γ response was defined as at least twice the CAT buffer treated wells. The spots formed in the presence of medium alone were subtracted from this to give the corrected SFC/10^6^ PBMC.

### ELISPOT assay (T-cell lines)

IFN-γ ELISPOT assays were performed using 96-well nitrocellulose plates that were pre-wet with ethanol, washed, and coated overnight with anti-human-IFN-γ mAb clone 1D1K (Mabtech) at 4°C. The plates were washed and blocked at RT with 10% FBS-RPMI for a minimum of 1 hr. Autologous B-lymphoblastoid cell lines (B-LCL, 5×10^4^ cells) were pulsed overnight with 1 µg/ml of pooled peptides representing CMDR Env or peptides generated from the Env-A244 proteolytic cleavage by CAT D, K, and L peptide-pulsed B-LCL were added the following day to 1×10^4^ expanded T cells and incubated at 37°C, 5% CO_2_ for 18–24 hrs. Production of IFN-γ by CD4^+^ T-cells was detected by addition of biotinylated anti-IFN-γ mAb Clone 7B-6 (Mabtech) for 2 hrs at 37°C. ELISPOT development consisted of a 1 hr incubation at RT with avidin horseradish peroxidase complex (Vectastain® ABC kit, Vector Labs, Burlingame, CA, USA) in PBS/0.05% Tween-20 buffer followed by washing with PBS, and incubation at RT with peroxidase substrate AEC for 4 min. Spots were counted with a C.T.L. analyzer and software (version 6.2, C.T.L. Analyzers). Results are expressed as spot-forming cells (SFC)/10^6^ PBMC. A positive IFN-γ response was defined as ≥3 times background (SFC)/10^6^ PBMC of B-LCL and T-cells with no peptide.

### Polychromatic ICS assay

Cryopreserved Env-specific T-cell lines were thawed, washed, and resuspended in RPMI-1640 with 10% NHS at 1×10^6^ cells/ml and then co-incubated for 4 hrs in the presence of 5×10^5^ B-LCL stimulated with pooled peptides (1 µg/ml) representing CMDR Env or peptides generated from the Env-A244 proteolytic cleavage by CAT D, K, and L. Anti-CD107a-FITC, anti-CD28/CD49 MAbs (BD Pharmingen), the protein transport inhibitors Monensin (BD Pharmingen) and Brefeldin A (Sigma-Aldrich) were included in the assay mix at set-up. Following the stimulation time, plates were washed, stained with Aqua Live/Dead (Invitrogen), washed, and resuspended in FACS wash buffer (0.5% BSA, 0.1% azide followed by surface staining with anti-CD14/CD19-Alexa700 (BD Pharmingen). Cells were simultaneously surface and intracellular stained with anti-CD4-ECD (Coulter), anti-IFN-γ-PB (eBioscience, San Diego, CA, USA), anti-TNF-α -PE-Cy7 and anti-MIP1β-PE (BD Pharmingen), anti-CD3-APC-H7, anti-CD8-PerCPCy5.5, and anti-IL2-APC (BD Biosciences). Cells were acquired on a BD LSR II cytometer (Becton Dickenson). Approximately 500,000 cells were acquired on a LSRII cytometer using BD FACSDiva software (BD Biosciences, San Jose, CA, USA) and analyzed using FlowJo software (Tree Star Inc, Ashland, OR, USA). Analysis and presentation of distributions was performed using SPICE version 5.1 [54].

## Supporting Information

Figure S1 **Env-A244 peptides derived by CAT K cleavage induce IFN-γ from the PMBC of RV144 volunteers.** PBMC from vaccine 144277 were stimulated with media, PHA, CMV peptides, CAT digestion buffers, or peptides derived from the CAT D and CAT K cleavage of Env-A244, and then analyzed for the generation of IFN-γ by an ELISPOT assay (top panel). Enlarged images of one of the triplicate ELISPOT wells are shown in the bottom panel. The spots obtained from 2×10^5^ PBMC with CAT K buffer and with peptides derived from the CAT K cleavage of Env-A244 are highlighted by arrows (bottom panel). The CAT K buffer had 2 spots, which translates to 10 spots/10^6^ cells. The Env-A244 CAT K digest had 8 spots, which translates to 40 spots/10^6^ cells.
(TIF)

Figure S2 **Env-A244 peptides derived by CAT K cleavage induce IFN-γ from the PMBC of RV144 volunteers.** PBMC from vaccine 144936 were stimulated with media, PHA, CMV peptides, CAT digestion buffers, or peptides derived from the CAT D and CAT K cleavage of Env-A244, and then analyzed for the generation of IFN-γ by an ELISPOT assay (top panel). Enlarged images of one of the triplicate ELISPOT wells are shown in the bottom panel. The spots obtained from 2×10^5^ PBMC with CAT K buffer and with peptides derived from the CAT K cleavage of Env-A244 are highlighted by arrows (bottom panel). The CAT K buffer had 0 spots, which translates to 0 spots/10^6^ cells. The Env-A244 CAT K digest had 3 spots, which translates to 15 spots/10^6^ cells.
(TIF)
