# Supplementary figures and images for: HIV-1 Envelope Resistance to Proteasomal Cleavage: Implications for Vaccine Induced Immune Responses
Source: PLoS One. 2012 Aug 6;7(8):e42579. doi: 10.1371/journal.pone.0042579 (PMC3412807; doi:10.1371/journal.pone.0042579)

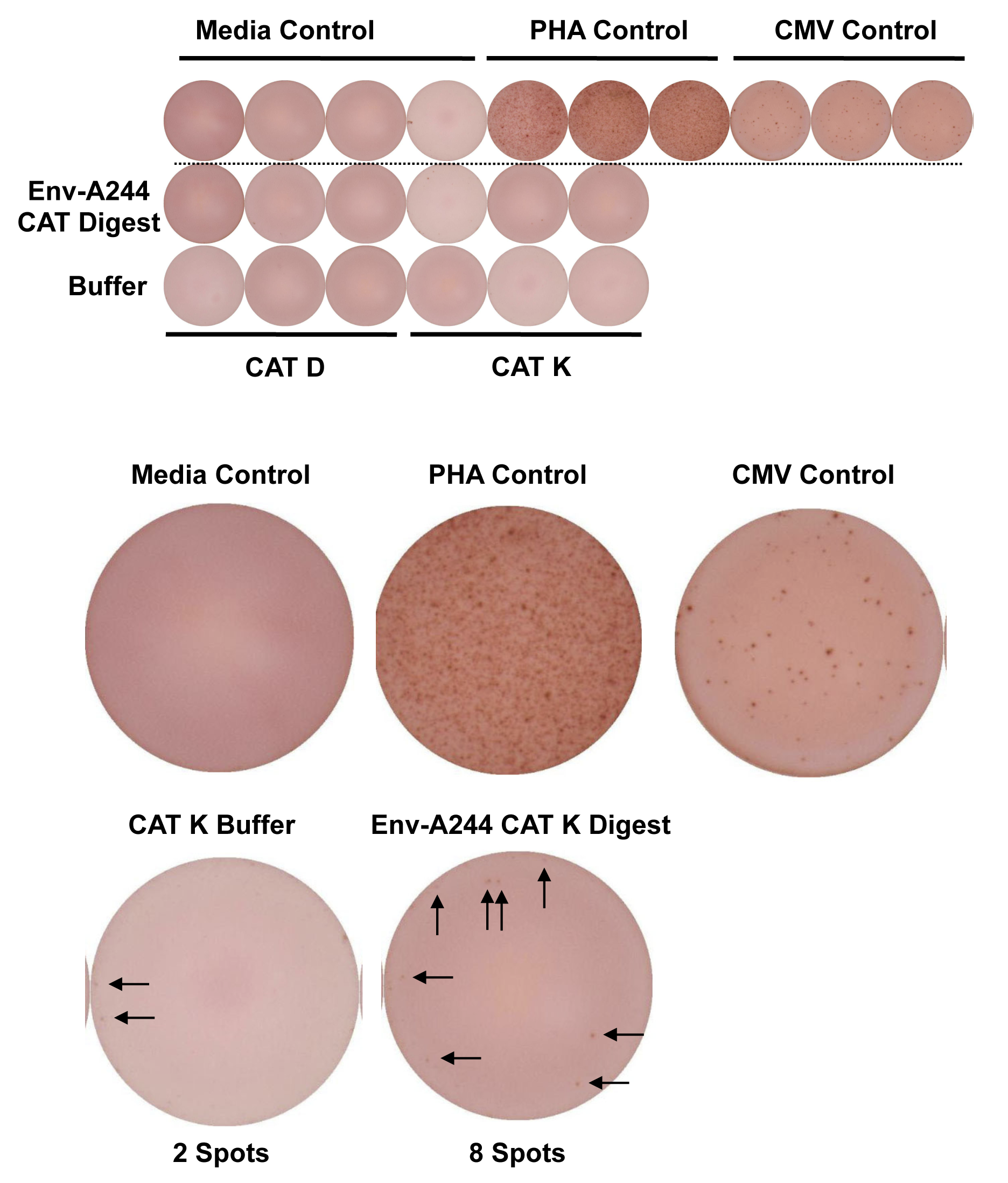

Supplement: Figure S1 — Env-A244 peptides derived by CAT K cleavage induce IFN-γ from the PMBC of RV144 volunteers. PBMC from vaccine 144277 were stimulated with media, PHA, CMV peptides, CAT digestion buffers, or peptides derived from the CAT D and CAT K cleavage of Env-A244, and then analyzed for the generation of IFN-γ by an ELISPOT assay (top panel). Enlarged images of one of the triplicate ELISPOT wells are shown in the bottom panel. The spots obtained from 2×10^5^ PBMC with CAT K buffer and with peptides derived from the CAT K cleavage of Env-A244 are highlighted by arrows (bottom panel). The CAT K buffer had 2 spots, which translates to 10 spots/106 cells. The Env-A244 CAT K digest had 8 spots, which translates to 40 spots/106 cells. (TIF) [file pone.0042579.s001.tif]

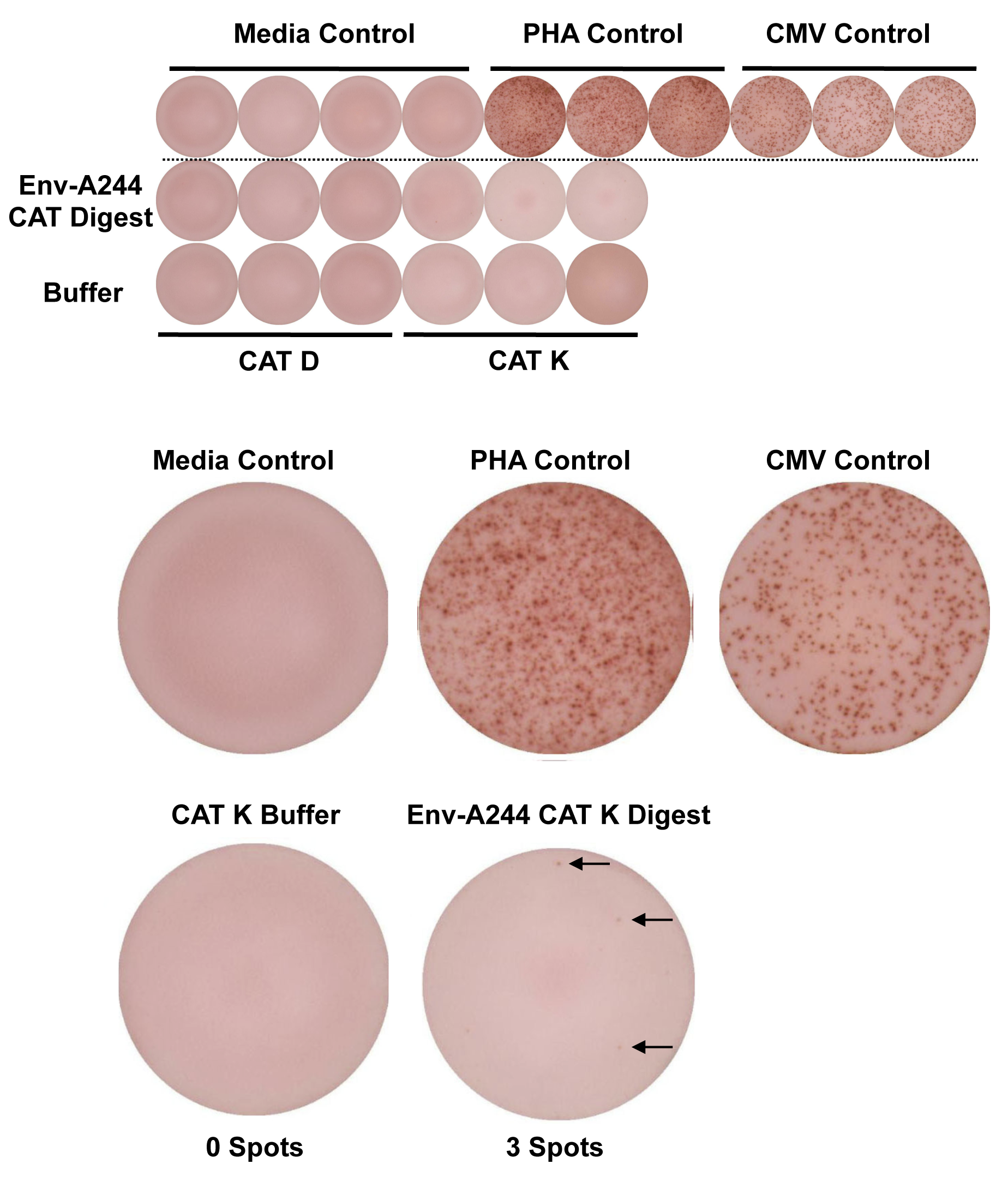

Supplement: Figure S2 — Env-A244 peptides derived by CAT K cleavage induce IFN-γ from the PMBC of RV144 volunteers. PBMC from vaccine 144936 were stimulated with media, PHA, CMV peptides, CAT digestion buffers, or peptides derived from the CAT D and CAT K cleavage of Env-A244, and then analyzed for the generation of IFN-γ by an ELISPOT assay (top panel). Enlarged images of one of the triplicate ELISPOT wells are shown in the bottom panel. The spots obtained from 2×10^5^ PBMC with CAT K buffer and with peptides derived from the CAT K cleavage of Env-A244 are highlighted by arrows (bottom panel). The CAT K buffer had 0 spots, which translates to 0 spots/106 cells. The Env-A244 CAT K digest had 3 spots, which translates to 15 spots/106 cells. (TIF) [file pone.0042579.s002.tif]
